# Modulation of GCN2 activity under excess light stress by osmoprotectants and amino acids

**DOI:** 10.1080/15592324.2022.2115747

**Published:** 2022-09-12

**Authors:** Ansul Lokdarshi, Albrecht G von Arnim, Teressa K Akuoko

**Affiliations:** a Department of Biology, Valdosta State University, Valdosta, GA, USA; b Department of Biochemistry & Cellular and Molecular Biology, University of Tennessee, Knoxville, TN, USA; c UT-ORNL Graduate School of Genome Science and Technology, the University of Tennessee, Knoxville, TN, USA

**Keywords:** GCN2, eIF2α, ROS, translation, abiotic stress, amino acid, osmoprotectants

## Abstract

The protein kinase GCN2 (General Control Nonderepressible2) and its phosphorylation target, the eukaryotic translation initiation factor (eIF)2α represent the core module of the plant’s integrated stress response, a signaling pathway widely conserved in eukaryotes that can rapidly regulate translation in response to stressful conditions. Recent findings indicate that the *Arabidopsis thaliana* GCN2 protein operates under the command of reactive oxygen species (ROS) emanating from the chloroplast under a variety of abiotic stresses such as excess light. To get deeper insights into the mechanism of GCN2 activation under excess light, we assessed the role of amino acids in view of the classic function of GCN2 as a sensor of amino acid status. Additionally, given that osmoprotectants can counteract ROS-related stresses, we tested their ability to mitigate GCN2 activity. Our results demonstrate that certain amino acids and osmoprotectants attenuate eIF2α-phosphorylation under excess light stress to some degree. Future investigations into the biochemical mechanisms of these natural compounds on GCN2 signaling activity will provide better insights into the GCN2-eIF2α regulation.

## Introduction

The integrated stress response is one of the most conserved stress response programs across all eukaryotes.^1–3^ The hallmark of the integrated stress response is the phosphorylation of the α-subunit of the heterotrimeric translation initiation factor eIF2 by the protein kinase, GCN2^1^, often resulting in suppression of general protein synthesis. Characteristic for GCN2 is its C-terminal tRNA-binding domain, which structurally resembles a histidyl-tRNA-synthetase.^4^ In animals and fungi, amino acid deprivation boosts the binding of uncharged tRNA to the histidyl-tRNA-synthetase domain, which activates the kinase.^5^ Upon phosphorylation, eIF2α-P binds to eIF2B, resulting in the formation of a stable eIF2α-P-GDP-eIF2B complex. eIF2B is a guanine nucleotide exchange factor that converts the inactive eIF2-GDP to the active eIF2-GTP. The decline in functional ternary complex (eIF2-GTP-tRNA(i)Met) results in inhibition of translation initiation.^5^ Some mRNAs that can remediate amino acid deprivation, escape this general translational repression owing to the presence of upstream reading frames in their mRNAs.^5^ GCN2 is therefore said to regulate the General Amino Acid Control (GAAC) in yeast^6^ and the amino acid response (AAR) in mammals.^7^

In plants GCN2 is the only known kinase that phosphorylates eIF2α.^8,9^ It is activated in response to a wide variety of stress conditions such as herbicides, UV light, cold and bacterial pathogens.^8–11^ The activation model of plant GCN2 via uncharged tRNA binding is based on its homology to the yeast GCN2^12^ and an *in vitro* study with Arabidopsis GCN2.^13^ Recent findings describe a new model of the Arabidopsis GCN2 activation by chloroplastic reactive oxygen.^14^ Specifically, stress conditions such as excess light (dark-to-light shift), herbicides, cold and salt affect the photosynthetic machinery to induce ROS hyper-accumulation. By an unknown mechanism, chloroplastic ROS then rapidly activates the cytosolic GCN2 leading to phosphorylation of eIF2α and subsequent adjustments to general protein synthesis.^14,15^ As chloroplastic ROS has mostly been linked to adjustments at the level of transcription (chloroplast-nucleus retrograde signaling),^16^ the findings presented in Lokdarshi et al., 2020a provide evidence of an additional, fast regulatory switch that targets cytosolic translation.^14^

The redox state of the plastoquinone pool (PQ/PQH2) playsan important role in the generation of ROS under a wide variety of stresses.^17,18^ Indeed, manipulation of the PQ/PQH2 pool with the herbicides, 3-(3,4-dichlorophenyl)-1,1-dimethylurea (DCMU) and 2,5-Dibromo-6-isopropyl-3-methyl-1,4-benzoquinone (DBMIB) led to the suppression of GCN2 activity under excess light, herbicide, cold and salt stress.^14,15^ Chloroplastic ROS regulates stress responses through different ways, including membrane damage under photo-oxidative stress.^18^ Regardless of how ROS exercise these effects, it is quite evident that the conventional model of plant GCN2 by uncharged tRNAs needs closer attention.

In keeping with the role of GCN2 as a sensor of amino acid status we examined the role of certain amino acids and osmoprotectants in mitigating GCN2 activity under excess light stress. Our results demonstrate that both of these agents mitigate the GCN2 activation triggered by excess light. These findings suggest a potential role of the natural compounds in chloroplast ROS management and/or regulation of the GCN2-eIF2α module under photo-oxidative stress.

## Results and discussion

*Zhang et al., 2008* showed that amino acid supplementation can suppress the growth defects in wild-type and *gcn2* mutant seedlings caused by herbicide treatments.^10^ Specifically, the lethal effects of chlorosulfuron (inhibits branched chain amino acid biosynthesis), glyphosate (inhibits aromatic amino acid biosynthesis) and IRL 1803 (inhibits histidine biosynthesis), can be mitigated with supplementation of the appropriate amino acids.^10^ Given the classic role of GCN2 in general amino acid control in yeast, we tested whether amino acids could mitigate Arabidopsis GCN2 activity under excess light stress.

**Figure 1.**
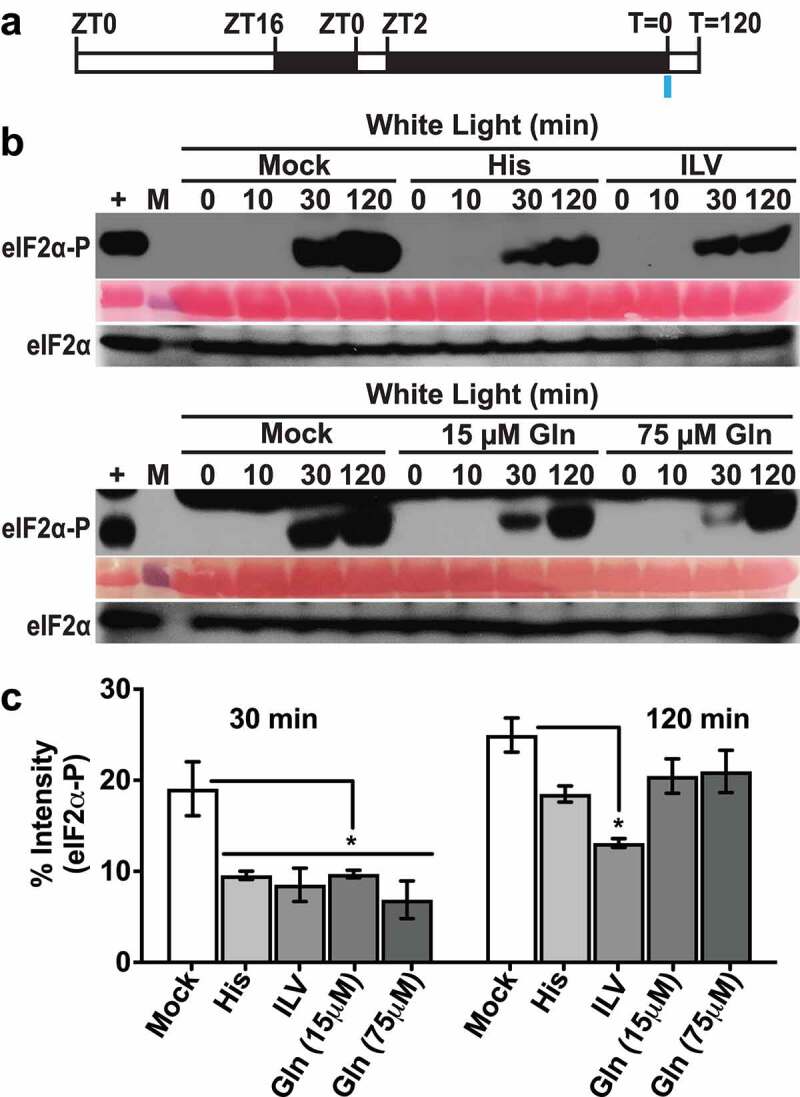
Effect of amino acid supplementation on GCN2 activation under excess light stress. (a) Schematic of the growth regimen. Seedlings were grown in a 16 h light (80µE): 8 h dark period, followed by a 24 h dark acclimation starting at zeitgeber time (ZT)2. The blue bar at T = 0 indicates the beginning of excess light treatment and the start of sampling. (b) Immunoblot showing the time course of eIF2*α* phosphorylation in 12-days-old wild-type Landsberg seedlings grown on media supplemented with either 1 mM Histidine (His) or a mixture of 1 mM each of isoleucine, leucine and valine (ILV), or 15 µM or 75 µM glutamine, and subjected to excess light stress as described in panel A. (Mock) Seedlings grown without any amino acid supplement and subjected to excess light stress. (+), arbitrary amount of total protein extract from herbicide treated Wt seedlings indicating unphosphorylated (eIF2α) or phosphorylated (eIF2α-P) protein (~38kDa); (10, 30, 120) sampling time in minutes. A ponceau stained blot showing Rubisco large subunit (~55kDa) is included as a loading control. M = Molecular weight marker. (c) Quantification of the eIF2α-P signal from immunoblots shown in (b). Error bars represent standard error of the mean from n ≥ 3 independent experiments. Welch’s unpaired *t* test P value *<0.05.

**Figure 2.**
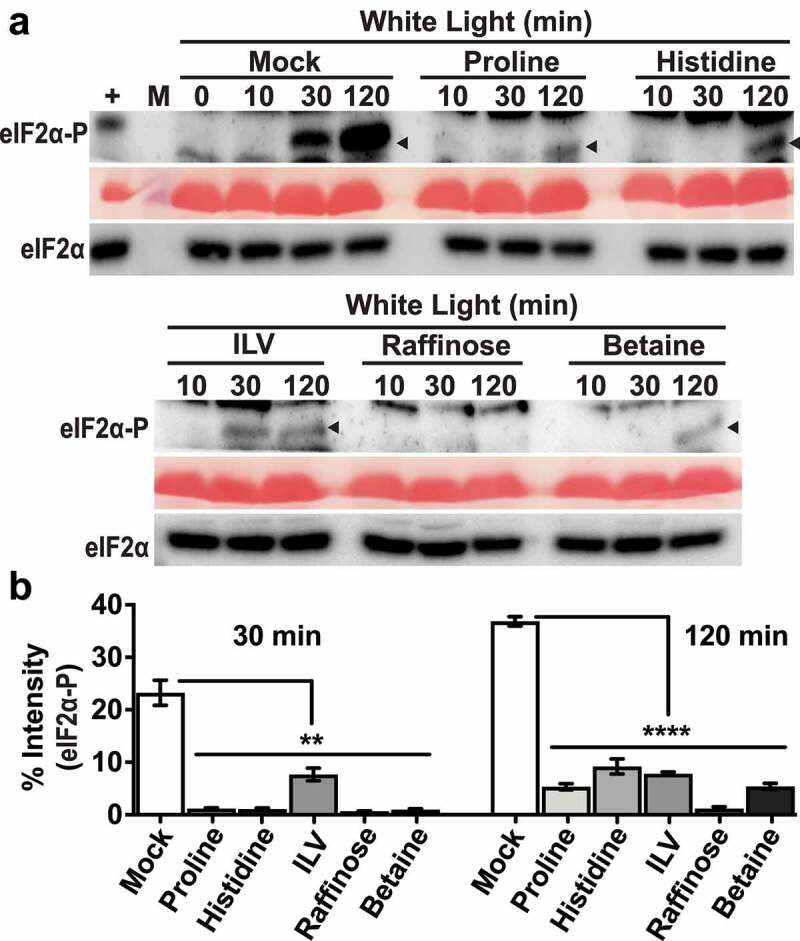
Amino acids and osmoprotectants mitigate eIF2α phosphorylation under excess light stress. (a) Immunoblot showing the time course of eIF2*α* phosphorylation in 12-days-old wild-type Landsberg seedlings subjected to excess light stress as described in Figure 1a. Thirty minutes prior to light exposure, seedlings were treated under green safe light with water only (Mock) or either of the following: 5 mM Proline, 5 mM Histidine, mixture of 5 mM isoleucine + leucine + valine (ILV), 5 mM Raffinose, 10 mM glycinebetaine (Betaine). Black arrow indicated the eIF2*α*-P band (~38kDa). For details see legend to Figure 1b. (b) Quantification of eIF2α-P signal from immunoblots shown in (a). Error bars represent standard error of the mean from four independent experiments. Welch’s unpaired t test P value **<0.005, ****<0.0001.

A low dosage (≤1 mM) was chosen for long-term amino acid feeding because higher concentrations of certain amino acids can cause growth defects.^19^ Based on eIF2α-P immunoblot analysis, different amino acids (glutamine, histidine and a mix of the three branched-chain amino acids), all inhibited GCN2 activity at 30 min of excess light stress (Figure 1a-c). The effect was more consistent with branched-chain amino acid mix as eIF2α-P levels remained significantly below mock treatment at 2 h of excess light stress. Total eIF2α levels remained similar to mock control in all conditions (Figure 1b). We also checked the effect of a shorter pre-treatment with amino acids on GCN2 activity (Figure 2a, b). Thirty-minute pre-treatment with histidine or branched-chain amino acid mix inhibited GCN2 activation at both 30 min and 2 h of excess light stress (Figure 2a, b). The inhibition of GCN2 activity was more prominent with histidine versus branched-chain amino acid mix at both the time intervals, while the total eIF2α levels remained similar under all conditions (Figure 2a, b).

**Figure 3.**
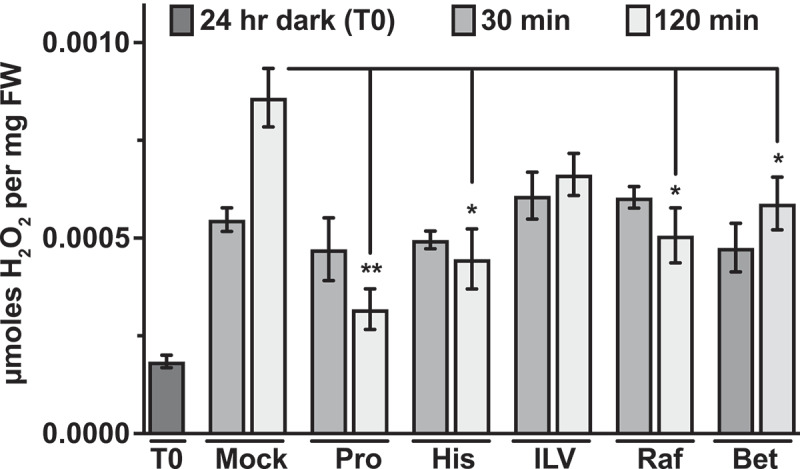
Effect of amino acid supplementation on GCN2 activation under excess-light stress. Relative H_2_O_2_ levels in 12-days-old wild-type seedlings subjected to excess-light stress as indicated in Figure 2. Mock (water), 5 mM Proline (Pro), 5 mM Histidine (His), mixture of 5 mM isoleucine + leucine + valine (ILV), 5 mM Raffinose (Raf), 10 mM glycinebetaine (Bet). Error bars represent standard error mean from three independent experiments. Welch’s *t*-test P-value ** <0.005, * <0.05 in comparison with Mock 120 min of excess-light.

In prior experiments, where GCN2 was activated by different herbicides that inhibit amino acids biosynthesis, amino acid supplementations had suppressed GCN2 activity.^9^ This effect had been attributed to the ability of the amino acids to rescue the toxicity of the herbicide, possibly by modulating the status of the charged tRNAs.^9^ Our observation does not contradict this interpretation but, given that we did not directly inhibit any amino acid synthesis pathway, it suggests that amino acids supplementation may suppress eIF2α-P through modes additional to the traditional model of GCN2 activation via uncharged tRNAs. For example, we previously showed that antioxidants such ascorbate and glutathione can suppress GCN2 activity under excess light stress.^14^ The antioxidant glutathione is derived from glutamine,^20^ and glutathione accumulates rapidly (within 20 seconds) after excess light stress in Arabidopsis.^21^ While changes in the status of tRNA charging within such a short time scale may be plausible, glutathione most likely suppresses the ROS buildup as a result excess light stress. To test the effect of amino acid supplementation on ROS levels under excess light stress, we performed H_2_O_2_ estimation from the same samples used for GCN2 activation discussed in Figure 2. Overall, we observed a general trend in suppression of H_2_O_2_ levels at the 2 h time interval versus mock (Figure 3). Taken together, we provide evidence supporting the role of amino acids in regulation of GCN2 activity under excess light stress via suppression of ROS.

Plant cells accumulate certain osmoprotectants (e.g., proline, glycine betaine, polyamines, di-and oligosaccharides) that aid in preventing cellular damage under a wide variety of stresses, including oxidative stress.^22,23^ For example, proline quenches ROS directly upon exogenous application on Arabidopsis roots^26^ and proline and raffinose may function both as an antioxidant and osmolyte.^24,27^ Consequently, osmoprotectants have been successfully used to enhance tolerance toward several abiotic stresses.^22^ Pretreatment of seedlings with proline and raffinose suppressed eIF2α-P upon excess light stress, at both 30 min and 2 h (Figure 2a, b). We also tested the osmolyte glycinebetaine^24,27^, which accumulates in higher plants under various stresses. Seedlings treated with betaine also showed similar suppression of eIF2α-P at both 30 min and 2 h of excess light stress (Figure 2a, b). Total eIF2α levels remained unchanged under all conditions (Figure 2a). Consistent with the inhibition of GCN2 activity, all the different osmoprotectants suppressed H_2_O_2_ accumulation at 2 h of excess light stress (Figure 3). Taken together, these data are consistent with the idea that GCN2 is also activated by ROS^14,15^ under excess light stress. Furthermore, the Arabidopsis GCN2 activation mechanism involves a step that can be influenced by metabolites that function as osmoprotectants or antioxidants.

Future investigations into understanding the mechanism of these natural metabolites on ROS accumulation and/or regulation of uncharged tRNA levels under excess light stress will yield further insights on the overarching theme of retrograde control of translation via the GCN2-eIF2α module.

## Material and methods

### Plant materials and growth conditions

Seeds of the *Arabidopsis thaliana* ecotype Landsberg *erecta* (Ler-0) were sterilized and stratified at 4°C for 2 days on ½-strength Murashige-Skoog (MS) salt plant medium (MP Biomedicals, cat # 2633024) containing 0.65% Phytoagar (Bioworld, cat # 40100072–2) without sucrose. Seeds were germinated and grown under a standard long-day cycle of 16 h light (white light, 80 ± 10 µE m^−2^s^−1^)/8 h dark at 22°C and 50% humidity.

### Stress treatments and amino acid/osmoprotectant supplementation

To expose seedlings to excess light, 12-days-old seedlings grown on horizontal petri plates (roots inside the medium) were dark-acclimated for 24 h, starting 2 hours after lights-on (Zeitgeber time 2, ZT2) as described in *Lokdarshi et al., 2020a*.^14^ After collecting a sample at the end of the 24 h dark acclimation (T = 0), plates were shifted back to white light (80 ± 10 µE m^−2^s^−1^) to induce excess light stress. For shorter pre-treatment with osmoprotectants and amino acids (pH 5.7), seedlings were submerged in either of the following reagents 30-min prior to end of 24 h dark acclimation: (L-proline (5 mM), Acros Organics cat# 147–85-3; betaine (10 mM), Sigma-Aldrich cat# B2629; D-(+)-raffinose pentahydrate (5 mM), Fluka cat# 17629–30-0), L-histidine (5 mM), Sigma cat# H9386; 5 mM each of L-isoleucine, Acros Organics cat# 73–32-5; L-leucine, MP Biomedicals cat# 194694; L-valine, Alfa Aesar cat# 72–18-4, or mock (water) control under safe green light. After incubation for 2–3 minutes, excess solution was drained off and the plates were placed back in dark for 30 minutes.

For treatment with amino acids in growth medium, seedlings were germinated and grown on amino acids with either of the following: L-histidine (1 mM); 1 mM each of L-isoleucine, L-leucine, L-valine; L-glutamine (15 µM or 75 µM), Alfa Aesar cat#56-85-9 for 12-days and excess light stress was performed as described above.

### Protein extraction, immunoblot analysis

Total protein was extracted as described in *Lokdarshi et al 2020a*.^14^ Briefly, 12-days-old seedlings were ground using a plastic pestle in a 1.5 ml tube with extraction buffer containing 25 mM Tris–HCl (pH 7.5), 75 mM NaCl, 5% (v/v) glycerol, 0.05% (v/v) Nonidet P-40, 0.5 mM EDTA, 0.5 mM EGTA, 2 mM DTT, 2% (w/v) insoluble PVP (Sigma P-6755), supplemented with 1 × protease and phosphatase inhibitor cocktail (Thermo-Fisher; cat# PIA32959).^25^ Total protein content was quantified by Bradford assay (Thermo-Fisher, cat# 23236). For immunoblot experiments, 50 µg of total protein was separated on a 12% (w/v) SDS-PAGE gel and immunoblot analysis with either phospho-eIF2α antibody (Cell Signaling, cat # 9712S; Lot # 18 and Lot #21) or polyclonal rabbit eIF2α antibody (a gift from Dr. Karen Browning, University of Texas, Austin) was performed as described in *Lokdarshi et al 2020a*^14^ and *Lokdarshi et al., 2015*.^28^ Quantification of eIF2α-P signal intensity and all statistical tests were performed using GraphPad Prism (ver. 9.4.1; GraphPad Software, Inc.).

### Hydrogen peroxide quantification

Hydrogen peroxide measurement using Amplex Red kit (Thermo-Fisher, cat# A22188) was performed as described in *Lokdarshi et al., 2020ab*^14,15^ with slight modification. Thirty milligrams of 12-days-old seedlings were ground to homogenous powder with a plastic pestle in a 1.5 ml tube sitting in liquid N_2_. Pulverized tissue was resuspended in 100 µl of 1X reaction buffer (Amplex Red kit) followed by vortexing for 1–2 minutes. After centrifugation of the tubes at 17,000 × g at 4°C for 4 min, the supernatant was used for H_2_O_2_ measurements as per the manufacturer’s instructions. Absorbance at 560 nm was measured on a microplate reader (Molecular Devices Spectramax M5).
